# Supplementary figures and images for: Methyltransferases of Riboviria
Source: Biomolecules. 2022 Sep 6;12(9):1247. doi: 10.3390/biom12091247 (PMC9496149; doi:10.3390/biom12091247)

Tree scale: 10

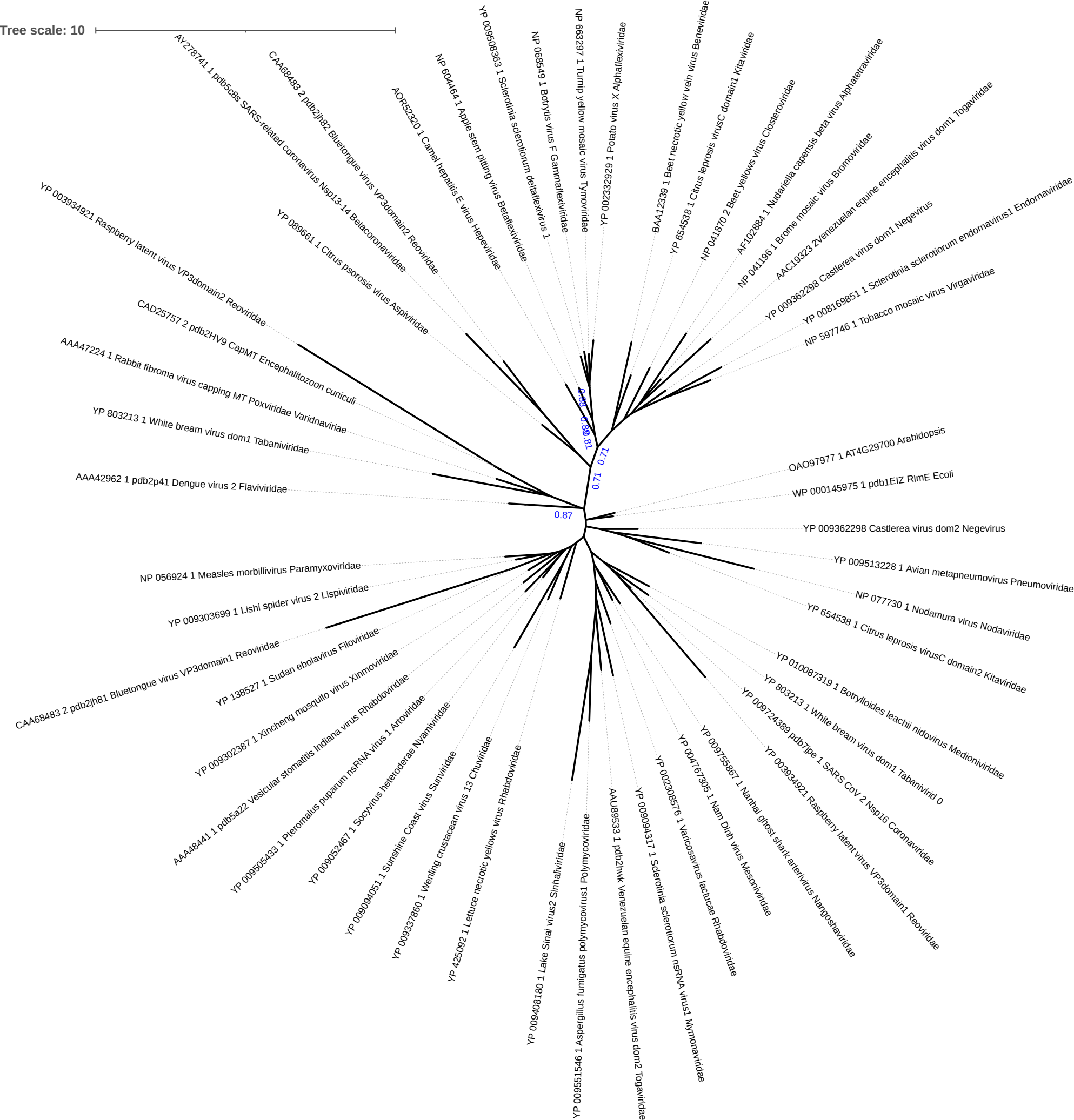

Supplement: Supplementary file 1 [file biomolecules-12-01247-s001.zip › Supplementary material File S4.pdf]
